# Detrended Fluctuation Analysis of Gait Cycles: A Study of Neuromuscular and Ground Force Dynamics

**DOI:** 10.3390/s25134122

**Published:** 2025-07-02

**Authors:** Soumya Prakash Rana, Maitreyee Dey

**Affiliations:** 1 School of Engineering, University of Greenwich, Medway Campus, Central Avenue, Chatham ME4 4TB, UK; 2 School of Computer Science and Digital Media, London Metropolitan University, 166-220 Holloway Road, London N7 8DB, UK; m.dey@londonmet.ac.uk

**Keywords:** detrended fluctuation analysis, human gait, electromyography, force-sensitive resistor, Tai Chi, neuromuscular control, human activity monitoring, wearable sensors

## Abstract

Gait analysis provides crucial insights into neuromuscular coordination and postural control, especially in ageing populations and rehabilitation contexts. This study investigates the complexity of muscle activation and ground reaction force patterns during gait by applying detrended fluctuation analysis (DFA) to electromyography (EMG) and force-sensitive resistor (FSR) signals. Data from a two-arm randomised clinical trial (RCT) supplemented with an observational control group were used in this study. Participants performed a single-task walking protocol, with EMG recorded from the tibialis anterior and lateral gastrocnemius muscles of both legs and FSR sensors placed under the feet. Gait cycles were segmented using heel-strike detection from the FSR signal, enabling analysis of individual strides. For each gait cycle, DFA was applied to quantify the long-range temporal correlations in the EMG and FSR time series. Results revealed consistent α-scaling exponents across cycles, with EMG signals exhibiting moderate persistence (α≈0.85–0.92) and FSR signals showing higher persistence (α≈1.5), which is indicative of stable and repeatable gait patterns. These findings support the utility of DFA as a nonlinear signal processing tool for characterising gait dynamics, offering potential markers for gait stability, motor control, and intervention effects in populations practising movement-based therapies such as Tai Chi. Future work will extend this analysis to dual-task conditions and comparative group studies.

## 1. Introduction

Human gait is a complex and dynamic process governed by integration of the neural, muscular, and skeletal systems [1]. It involves the coordination of repetitive locomotor cycles, with temporal and spatial gait parameters reflecting the stability, rhythm, and adaptability of an individual’s movement patterns. Disruptions in gait are common with ageing, and are often associated with increased risk of falls, mobility limitations, and loss of independence [2,3]. Recent advancements in ultra-wideband (UWB) positioning systems have introduced a promising wireless alternative for gait monitoring, offering high-precision motion tracking without requiring direct physical contact [4]. Several studies [5,6,7,8,9] have demonstrated the viability of UWB for both indoor localisation and remote gait assessment, positioning it as a powerful tool for real-time markerless motion analysis. In parallel with these hardware innovations, signal processing methodologies for gait analysis have also advanced significantly, enabling more nuanced interpretation of physiological signals.

The ancient Chinese martial art of Tai Chi has gained prominence as a mind–body exercise for improving balance, flexibility, and neuromuscular coordination. Its slow and deliberate movements together with its emphasis on posture and weight shifting have been shown to enhance postural control and functional mobility [10]. As a low-impact and accessible form of physical activity, Tai Chi has demonstrated efficacy in reducing fall risk and improving gait parameters among older adults [11,12]; however, the physiological mechanisms underlying these improvements are not yet fully understood. To investigate the neuromechanical effects of Tai Chi on human gait, objective and sensitive measurement techniques are essential. Surface electromyography (EMG) provides insight into muscle activation patterns during movement [13], while force-sensitive resistor (FSR) sensors are able to capture plantar pressure and foot–ground interaction dynamics [14]. Together, these signals offer a comprehensive view of both the neuromuscular and biomechanical components of gait. Recent advances in signal processing and artificial intelligence (AI) have enabled deeper analysis of physiological data beyond conventional spatiotemporal parameters. One such method is detrended fluctuation analysis (DFA), a nonlinear approach that quantifies long-range correlations and fractal scaling properties in time series data [15,16]. DFA is particularly useful for analysing non-stationary signals such as EMG and FSR, as it reveals the temporal complexity and self-similarity inherent in physiological control systems.

Based on prior studies indicating improved postural control and neuromuscular regulation following Tai Chi practice, it was hypothesised that participants with higher Tai Chi experience would exhibit elevated DFA α values, reflecting enhanced gait stability and more consistent neuromuscular coordination compared to individuals without Tai Chi training.

## 2. Related Work

Numerous studies have investigated human gait variability and its relationship with neuromuscular function, fall risk, and ageing. Hausdorff et al. [17] demonstrated that healthy gait exhibits fractal-like fluctuations which become more random in pathological conditions. Similarly, Näf et al. [18] found reduced gait complexity in patients with Parkinson’s disease. These findings have motivated the application of nonlinear tools such as DFA to gait analysis. Tai Chi has received increasing research attention due to the benefits it confers in terms of balance and gait in older adults. Wayne and Kaptchuk [19] reviewed the methodological strengths and limitations of Tai Chi research, encouraging more physiological studies. Studies by Li et al. [11] and Wu [20] have reported that Tai Chi practice enhances proprioception and lower-limb strength, contributing to improved gait stability. Manor et al. [21] provided evidence of increased physiological complexity in Tai Chi practitioners through non-invasive cardiac measurements. In terms of signal acquisition, EMG and pressure sensors have been widely used to characterise gait. Winter [22] outlined foundational principles for EMG interpretation during walking. Meanwhile, advancements in wearable pressure sensor technology have enabled portable and continuous monitoring of gait [23,24]. These tools are increasingly integrated into artificial intelligence (AI)-based systems for rehabilitation and fall prevention [25]. The use of DFA in gait analysis has grown steadily. Hausdorff et al. [17] showed that stride interval fluctuations are not random but exhibit long-range correlations. More recent work by Phinyomark et al. [26] reviewed nonlinear features in EMG signal classification, including DFA. Chiang et al. [27] applied DFA to investigate stride consistency under dual-task walking, while Kang and Dingwell [28] used DFA to study the effect of visual feedback on gait variability. DFA has also been applied in rehabilitation contexts; Lamoth et al. [29] evaluated post-stroke gait dynamics, while Costa et al. [30] highlighted DFA’s capacity to detect pathological signal features at multiple scales. These studies support DFA’s utility in assessing movement complexity, especially when used in combination with EMG and pressure data.

### Problem Statement and Contributions

Despite increasing interest in Tai Chi as a therapeutic intervention to improve balance and motor coordination, to date only limited research has explored the detailed complexity of gait in Tai Chi practitioners compared to non-practitioners. In particular, the strength and structure of long-range temporal correlations in gait-related signals captured using nonlinear methods such as DFA have not been extensively investigated across distinct training levels. Most existing studies focus on average gait parameters, lacking both cycle-level granularity and neuromechanical interpretation of variability across diverse groups such as control, Tai Chi practitioners, and master-level practitioners. This leaves a gap in understanding how regular physical practice such as Tai Chi influences stride stability and neuromuscular control as captured by synchronised EMG and FSR signals.

This study contributes to the literature in several key ways. First, it applies DFA to synchronised EMG and FSR signals segmented by gait cycles, enabling high-resolution analysis of stride dynamics. Second, it compares gait complexity across three distinct groups (control, Tai Chi, master) using leg-specific signal analysis, thereby offering new insights into the neuromuscular and mechanical adaptations associated with Tai Chi practice. Finally, it introduces a cycle-by-cycle DFA framework that can serve as a foundation for future AI-based gait classification models and rehabilitation monitoring systems.

The rest of this paper is structured as follows: Section 3 presents the data acquisition methodology, describing the sensor setup, FSR sensor signal processing for gait cycle identification and DFA computation, EMG sensor signal processing for muscle’s electrical activity and DFA computation, and interpretation process; Section 4 outlines the results and discussion; finally, Section 5 and Section 6 provide conclusions and directions for future research.

## 3. Methodology

**Figure 1:**
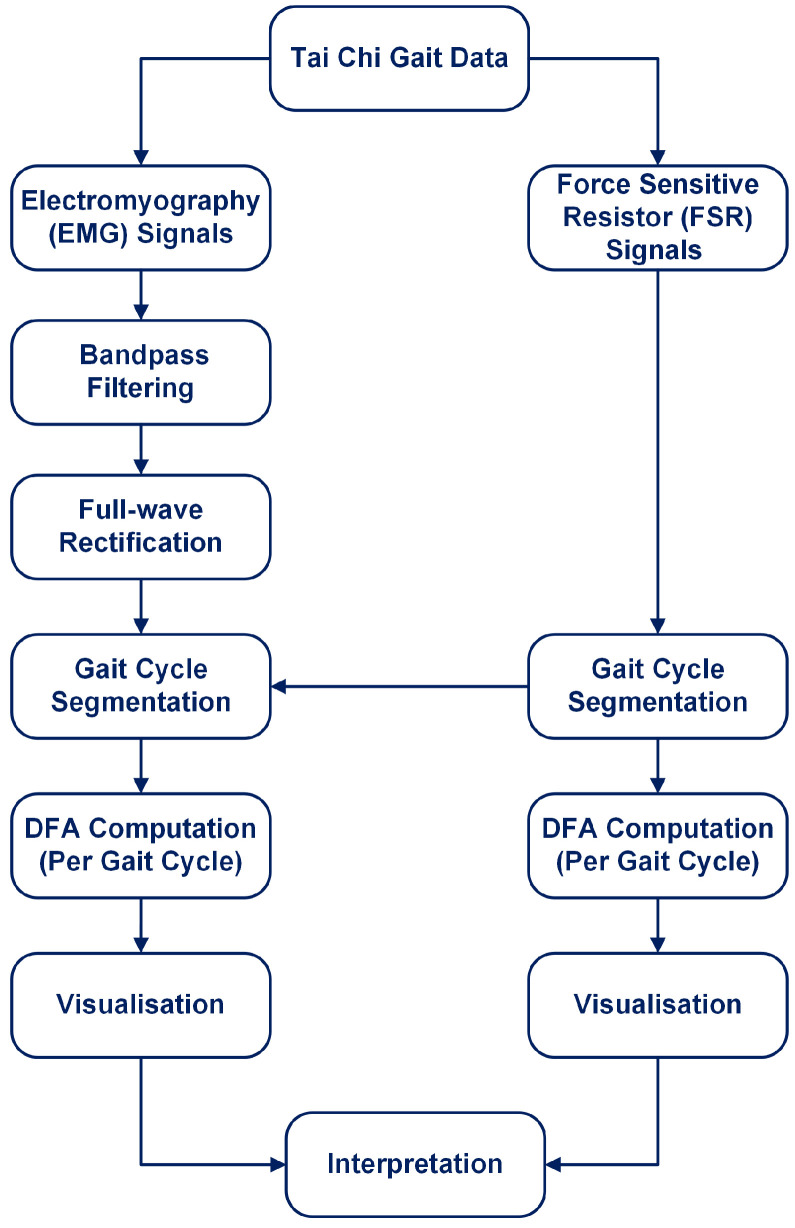
Overview of the analysis workflow: EMG and FSR signals are extracted, preprocessed, and segmented by gait cycles, then DFA is applied to each segment, followed by visualisation and interpretation of the results.

The overall workflow for this study is illustrated in Figure 1. The process begins with the Tai Chi Gait Dataset, from which both EMG and FSR sensor signals are extracted. FSR signals are used to segment gait cycles by detecting heel strikes. EMG signals are preprocessed using bandpass filtering and full-wave rectification. After identifying gait cycles, both EMG and FSR signals are segmented accordingly. DFA is applied directly to full-length EMG and FSR signals segmented on a per-gait cycle basis. The entire gait cycle (stance and swing phases combined) is analysed without isolating specific gait subphases. This approach allows for the capture of intra-cycle dynamics across repeated strides, ensuring that both neuromuscular activation patterns and ground contact forces are fully represented for each cycle. DFA is applied to each segmented signal on a per-cycle basis to compute the scaling exponent α, which quantifies the temporal correlation structure of the signal. Finally, the results are visualised and interpreted to assess gait regularity and neuromuscular control. Each of these steps is detailed in the subsequent subsections.

### 3.1. Dataset Description and Participant Groups

**Figure 2:**
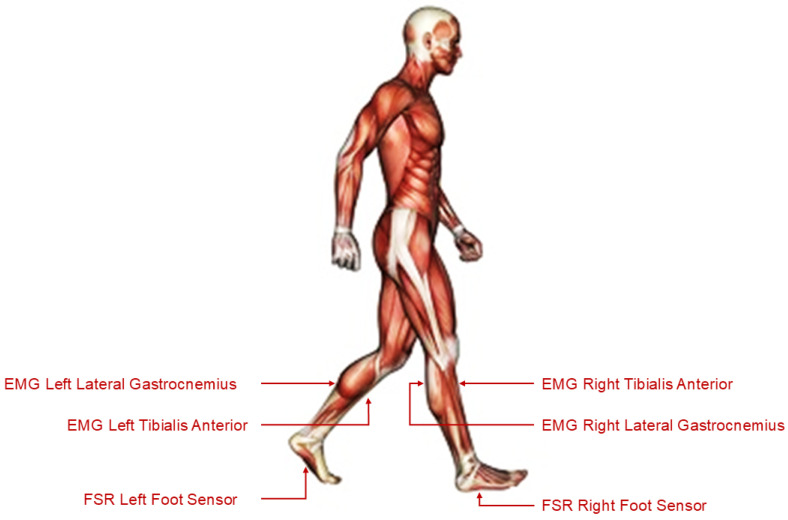
Sensor placement on the lower limbs used for EMG and FSR acquisition during gait trials.

The dataset used in this study originates from the Tai Chi Gait Database, which is publicly available on PhysioNet [16,31,32]. This dataset contains synchronised multimodal recordings of human gait acquired using FSR sensors and surface EMG electrodes. Data were collected during standardised walking trials from participants categorised into three distinct groups: (a) Control, consisting of individuals with no prior Tai Chi training; (b) Tai Chi Practitioners, consisting of individuals with non-expert Tai Chi experience; and (c) Tai Chi Masters, consisting of highly trained Tai Chi experts. Each subject’s gait cycle was recorded at a high sampling frequency of $f_{s}=$ 1500 Hz. The dataset was stored in WFDB format, and included EMG signals from the Tibialis Anterior and Lateral Gastrocnemius muscles (on both the left and right legs) alongside FSR data from the soles of both feet. These sensor placements are illustrated in Figure 2, which depicts the anatomical locations targeted for signal acquisition. For processing and model training, the recordings were segmented into uniform windows of 15 s each in order to facilitate consistent feature extraction and time series modelling.

### 3.2. FSR Sensor Signal Processing and DFA Computation

FSR data were collected using in-shoe pressure sensors embedded beneath the right and left feet. These sensors recorded vertical ground reaction forces as participants walked on a level surface. Each participant was equipped with two FSR sensors, one positioned under each foot. The FSRs were standard capacitive foot-switch sensors integrated into in-shoe inserts, and were capable of capturing vertical ground reaction forces with millisecond temporal resolution. Each sensor captured the timing and intensity of foot–ground contact events sampled at 1500 Hz. This high sampling rate ensured precise detection of gait events such as heel strikes and toe-offs, which are fundamental for analysing step dynamics. The FSR signals reflect the load and contact pressure under the feet throughout the gait cycle. Peaks in the signal indicate the stance phase (when the foot is on the ground), while troughs correspond to the swing phase. These patterns are used to determine gait phase transitions and detect walking regularity or variability across cycles. To ensure consistency across participants and reduce inter-subject variability, the FSR signals were normalised using min–max scaling $x_{\mathrm{norm}}=\frac{x-\min(x)}{\max(x)-\min(x)}$. This transformation compresses all signal values into the range [0, 1], allowing for meaningful comparisons of signal dynamics across subjects with different foot pressure magnitudes. As the dataset was obtained from a publicly available repository, detailed information regarding factory calibration of the FSR sensors is not explicitly available. However, based on standard data acquisition protocols used in such systems it is reasonable to assume that the sensors were factory-calibrated prior to data collection.

#### 3.2.1. Gait Cycle Segmentation

Accurate identification of gait cycles is essential for analysing intra-cycle dynamics. A dynamic threshold was computed as the average of the minimum and maximum values of the normalised right-foot FSR signal. Heel strike events were identified as rising edges crossing this threshold. Consecutive heel strikes defined the start and end points of individual gait cycles. A minimum inter-strike interval of 0.5 s was imposed to eliminate spurious detections caused by signal noise or within-step fluctuations.

#### 3.2.2. DFA Computation

DFA was employed to quantify long-range temporal correlations in each segmented FSR signal. This nonlinear technique is particularly well suited to analysing non-stationary physiological time series such as those associated with human gait. The process begins by integrating the original signal $x_{i}$ to obtain a cumulative sum Y(k), defined as follows:

(1) $$Y\left(k\right)=\sum_{i=1}^{k}\left(x_{i}-\bar{x}\right)$$

where $\bar{x}$ is the mean of the signal. The integrated signal Y(k) is then divided into non-overlapping windows of equal length *n*. Within each window, a linear polynomial is fitted to represent the local trend $Y_{\mathrm{trend}}\left(k\right)$, which is subtracted to de-trend the signal. The root mean square (RMS) fluctuation F(n) for window size *n* is calculated as

(2) $$F\left(n\right)=\sqrt{\frac{1}{N}\sum_{k=1}^{N}{\left(Y\left(k\right)-Y_{\mathrm{trend}}\left(k\right)\right)}^{2}},$$

where *N* is the total number of data points. By plotting logF(n) against logn, a linear relationship is typically observed, with the slope of this line corresponding to the DFA scaling exponent α. This exponent characterises the strength of long-range correlations in the signal: α≈0.5 indicates uncorrelated white noise, α>0.5 suggests persistent long range correlations, and α<0.5 implies anti-persistent behaviour. The DFA results were visualised using log–log plots for each gait cycle. These plots included all relevant FSR signals (right and left foot), with the corresponding α values displayed in the legends. Higher α values indicate persistent and consistent foot–ground interaction patterns, often associated with stable and rhythmic gait.

### 3.3. EMG Sensor Signal Processing and DFA Computation

EMG data were collected using surface electrodes placed over the tibialis anterior and lateral gastrocnemius muscles of both legs. These signals capture the electrical activity generated by muscle fibres during the contraction and relaxation phases of walking. Signals were sampled at 1500 Hz to capture high-resolution information about muscle activation patterns. The EMG signals represent the underlying neuromuscular activation required to produce and modulate movement. High-amplitude EMG bursts typically correspond to phases of active muscle contraction, such as during push-off or foot clearance in gait. Analysis of these patterns allows for assessment of motor control and coordination.

#### 3.3.1. Bandpass Filtering and Full-Wave Rectification

To remove low-frequency motion artefacts and high-frequency electrical noise, a fourth-order Butterworth bandpass filter with cutoff frequencies of 20 Hz and 450 Hz was applied to each EMG channel. This filtering preserves the physiological frequency range of interest while minimising signal distortion. The filter was applied using a zero-phase forward-backward method to prevent phase shifts in the signal.

After filtering, EMG signals were rectified by taking their absolute value $x_{\mathrm{rect}}=\left|x_{\mathrm{filtered}}\right|$. This step converts all signal values to positive values, facilitating the extraction of amplitude envelopes and improving interpretability for time domain analyses such as DFA. To standardise EMG amplitude across individuals and muscles, each rectified EMG signal was normalised to its maximum value observed during the trial $x_{\mathrm{norm}}=\frac{x_{\mathrm{rect}}}{\max(x_{\mathrm{rect}})}$, allowing for comparison of signal structure without being confounded by raw amplitude differences due to electrode placement or muscle strength variability.

#### 3.3.2. Gait Cycle Segmentation

The gait cycle boundaries identified from the right-foot FSR signal were used to segment the EMG signals. This ensures that EMG activity is analysed in direct correspondence with biomechanical events, enabling the study of neuromuscular control within each individual gait cycle.

#### 3.3.3. DFA Computation

The same DFA procedure described for FSR signals (in Section 3.2.2) was applied to each EMG segment. The resulting α exponent captures the degree of complexity and long-range temporal correlation in the muscle activation pattern. Higher α values typically reflect more predictable rhythmic muscle activity, while lower values may indicate greater variability or neuromuscular dysfunction. DFA results were plotted for each EMG channel across individual gait cycles. Separate figures were produced for the right and left legs, each showing the tibialis anterior and lateral gastrocnemius EMG along with the corresponding foot FSR signal. These visualisations help with interpreting the relationship between muscle activation and gait dynamics.

### 3.4. ANOVA and Interpretation

To statistically assess group-wise differences in gait complexity, a one-way ANOVA test was performed on the DFA α values extracted from the EMG and FSR signals of both the left and right legs. Specifically, signals from FSR sensors on the tibialis anterior, lateral gastrocnemius, and foot were analysed for each gait cycle and grouped by participant classification (Control, Tai Chi, and Master). ANOVA was used to evaluate whether the mean α values differed significantly across these groups, and post hoc multiple comparison tests were applied when significant effects (p<0.05) were observed. DFA results were computed for both limbs in order to ensure symmetry and completeness.

In this study, the FSR and EMG signals were used in a complementary manner to investigate both biomechanical and neuromuscular aspects of gait. FSR data provide accurate temporal markers for gait events and quantify the consistency of foot-ground interactions, offering insights into overall gait rhythm and stability; on the other hand, EMG data reveal the underlying muscle activation patterns responsible for producing those movements, capturing the neuromuscular control dynamics.

By applying DFA to both signal types within the same gait cycles, it was possible to explore how the regularity and complexity of muscle activation relate to those of physical foot contact with the ground. A high DFA scaling exponent (α) in FSR signals indicates rhythmic and stable walking, while high α in EMG reflects the smoothness and coordination of muscle contractions. Together, these metrics allow for a holistic understanding of gait dynamics, enabling comparison across conditions such as Tai Chi training, control, or ageing-related decline.

## 4. Experimental Results and Analysis

This study utilised gait and EMG data from a hybrid dataset comprising a two-arm RCT and an observational comparison group [16,31,32]. The RCT involved 60 healthy Tai Chi-naïve adults aged 50–79, who were randomly assigned to either a six-month Tai Chi training program or a usual care group, with assessments at baseline, 3 months, and 6 months. Additionally, 27 experienced Tai Chi practitioners (with >5 years of practice) formed an observational group that was assessed at baseline only. Participants walked at their preferred pace under two conditions: a single task (10 min of walking) and a dual task (90 s of walking while performing verbal serial subtractions). Gait events were captured using wireless FSR foot-switches placed under the toes and heel, and gait speed was calculated from the total distance covered. Surface EMG data were recorded bilaterally from the tibialis anterior and lateral gastrocnemius muscles using a Noraxon system, while gait data were acquired using the ME6000 system from Mega Elektronika, Inc., New Brunswick, NJ, USA. Both EMG and FSR signals were sampled at 1500 Hz, with EMG signals low-pass filtered at 500 H to enable synchronised high-resolution analysis of muscle activity and gait timing.

### 4.1. Synchronised FSR and EMG Sensor Signal Behaviour

**Figure 3:**
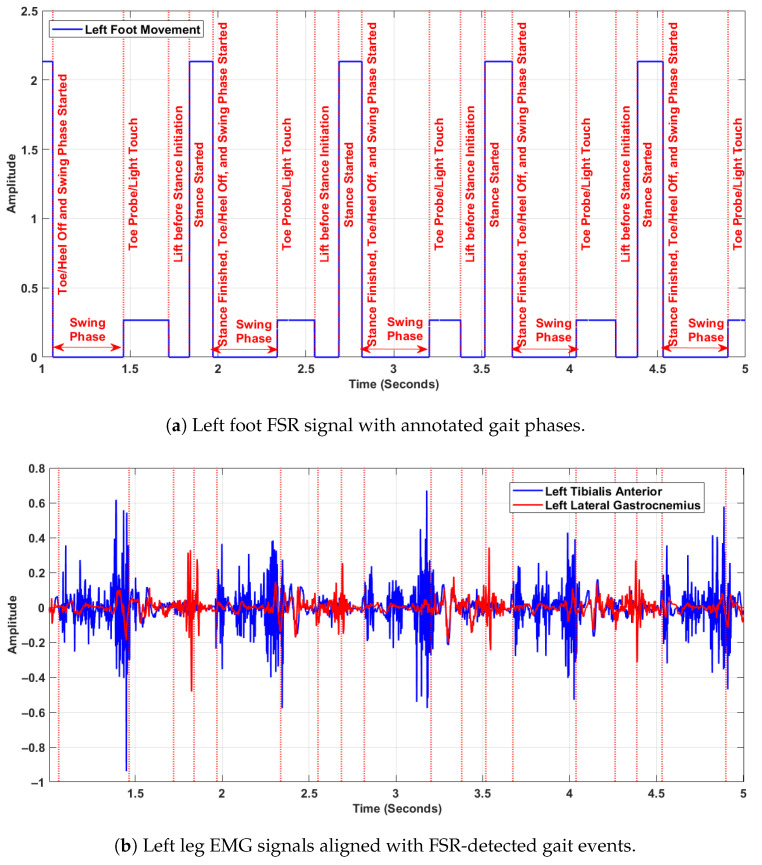
Synchronised FSR and EMG signals from the left leg, showing alignment between gait events and muscle activity.

Figure 3 presents synchronised data from the left foot FSR signal and left leg EMG signals, illustrating the temporal alignment of biomechanical gait phases with neuromuscular activation patterns. The top Figure 3a shows the annotated FSR signal, while the bottom Figure 3b displays the raw EMG recordings from the left tibialis anterior and left lateral gastrocnemius muscles. All signals are synchronised in time and were sampled at 1500 Hz. In the upper plot (Figure 3a), distinct gait events are annotated within each stride cycle, including phases such as toe probe/light touch, lift before stance initiation, stance started, and stance finished/toe-off. The swing phase is explicitly marked between successive stance phases. These events were extracted from the FSR signals based on amplitude transitions, and are used as reference markers for gait segmentation. In the lower plot (Figure 3b), the EMG activity reveals clear modulation across the gait cycle. The tibialis anterior shows increased activity during the swing phase, particularly during foot clearance and pre-heel-strike, reflecting its role in dorsiflexion. The lateral gastrocnemius shows greater activation just before and during the stance phase, indicating its function in plantarflexion and propulsion. These muscle activations align temporally with the biomechanical transitions identified in the FSR signal. Together, the two panels demonstrate the coordination between muscular control and foot–ground interaction during walking. This synchronised representation is essential for understanding intra-cycle dynamics, and validates the use of FSR-based segmentation to interpret neuromuscular behaviour in gait analysis. The purpose of aligning EMG signals with FSR-detected gait events was to ensure precise correspondence between muscle activation and biomechanical gait phases within each stride. This synchronisation allowed DFA to be consistently applied to matched EMG and FSR segments, enabling analysis of neuromuscular complexity during complete gait cycles rather than isolated phases.

### 4.2. DFA-Based Gait Analysis in Control Participants

**Figure 4:**
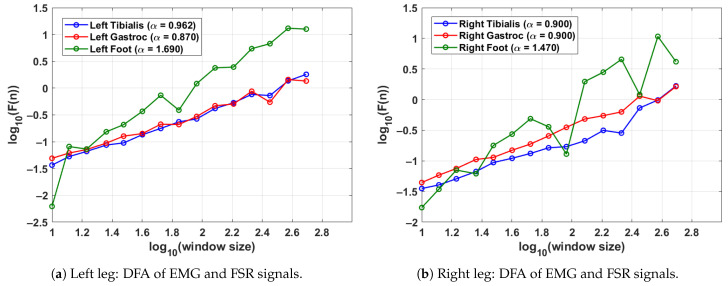
DFA results from a control subject, showing the scaling exponents for EMG and FSR signals in a single gait cycle. EMG and FSR signals were processed separately for each leg using leg-specific gait segmentation, while the gait cycle was segmented using the FSR for the left and right feet.

Figure 4 shows the DFA results for a single gait cycle of both the left leg (shown in Figure 4a) and right leg (shown in Figure 4b) in a control subject. The DFA plots illustrate the fractal scaling behaviour of surface EMG signals from the tibialis anterior and lateral gastrocnemius muscles as well as the FSR signal from the corresponding foot. Gait cycles were segmented using heel strike events identified from each leg’s respective FSR signal, ensuring side-specific temporal alignment. The scaling exponent α derived from the slope of the log–log plot quantifies the long-range correlation structure of each signal. For the left leg (shown in Figure 4a), the tibialis anterior and gastrocnemius show α values of 0.962 and 0.870, respectively, indicating moderately persistent muscle activation patterns. The corresponding FSR signal exhibits a higher α of 1.690, reflecting strong rhythmicity and regular foot–ground contact. For the right leg (shown in Figure 4b), both EMG channels yield balanced α values of 0.900 while the FSR shows a slightly lower but still persistent value of 1.470. These results demonstrate the consistency of motor control and gait rhythm in healthy individuals. Moreover, the differences between the EMG and FSR α values highlight the complementary roles of neuromuscular activation and mechanical foot dynamics in gait analysis.

### 4.3. DFA-Based Gait Analysis in Tai Chi Practitioner Participants

**Figure 5:**
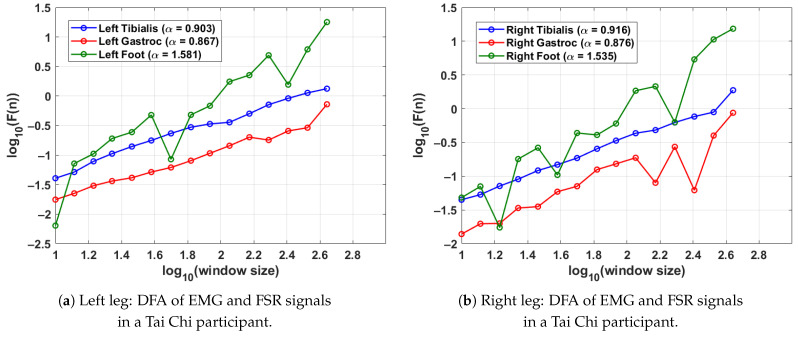
DFA plots for a Tai Chi participant, showing EMG and FSR scaling behaviour across a single gait cycle. Higher FSR α values reflect rhythmic and stable walking, while the EMG α values demonstrate coordinated neuromuscular activation. The gait cycle was segmented using the FSR for the left and right feet.

Figure 5 shows the DFA results from a single gait cycle of a participant in the Tai Chi group. As in the previous analyses, the scaling exponent α was computed from EMG signals of the tibialis anterior and lateral gastrocnemius muscles along with the FSR signal from the corresponding foot. Each leg’s gait cycle was segmented using its respective FSR signal to preserve temporal precision. For the left leg (shown in Figure 5a), the DFA exponents are α=0.903 (tibialis anterior), α=0.867 (gastrocnemius), and α=1.581 (FSR), reflecting consistent neuromuscular control and rhythmic stride execution. In the right leg (shown in Figure 5b), the tibialis anterior and gastrocnemius yield α=0.916 and α=0.876, respectively, while the right foot FSR signal shows an exponent of α=1.535. These results indicate that the Tai Chi participant in this case demonstrates well-regulated muscle activation patterns and stable gait dynamics in both limbs. Compared to control participants, slightly higher FSR α values suggest enhanced stride regularity, which may reflect long-term motor control benefits associated with Tai Chi practice.

### 4.4. DFA-Based Gait Analysis in Tai Chi Master Participants

**Figure 6:**
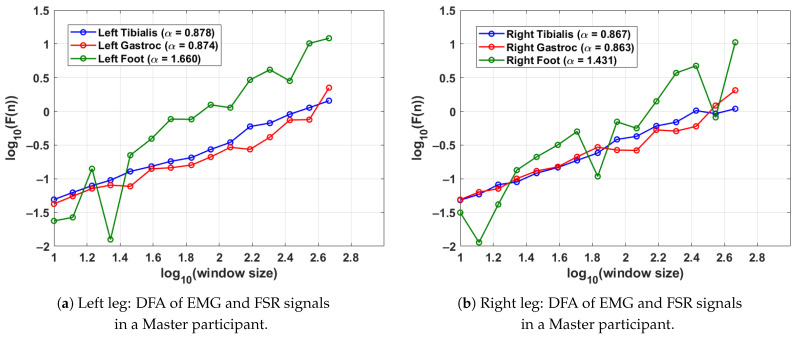
DFA results for a Master Tai Chi participant, illustrating the fractal properties of EMG and FSR signals during a single gait cycle. Higher α values in FSR indicate stable and rhythmic stride patterns, while consistent EMG α values reflect balanced neuromuscular activation. The gait cycle was segmented using the FSR for the left and right feet.

Figure 6 presents the DFA results for a single gait cycle from both the left leg (shown in Figure 6a) and right leg (shown in Figure 6b) of a Master participant with long-term Tai Chi experience. The analysis captures the fractal scaling behaviour of surface EMG signals from the tibialis anterior and lateral gastrocnemius muscles as well as FSR data from the respective foot. Gait segmentation was performed individually for each leg using heel strike events from the corresponding FSR signal. For the left leg (shown in Figure 6a), the tibialis anterior and gastrocnemius muscles exhibit respective DFA scaling exponents of α=0.878 and α=0.874, indicating moderately persistent and coordinated neuromuscular activity. The FSR signal for the left foot shows a higher exponent of α=1.660, suggesting highly regular and rhythmic foot–ground contact characteristic of refined gait patterns. Similar values are observed for the right leg (shown in Figure 6b); the tibialis anterior and gastrocnemius yield α=0.867 and α=0.863, respectively, with the FSR signal for the right foot showing α=1.431. These findings imply balanced neuromuscular control and stride regularity across both legs. The results collectively demonstrate consistent well-regulated gait dynamics in this Master participant, supporting the hypothesis that long-term Tai Chi practice enhances both muscular coordination and locomotor stability.

### 4.5. Comparison of DFA α Values Across Groups

**Figure 7:**
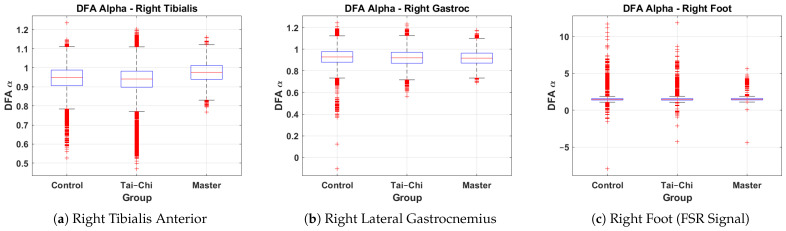
Group-wise comparison of DFA α values for the (**a**) right tibialis anterior, (**b**) right lateral gastrocnemius, and (**c**) right foot signals across the Control, Tai Chi, and Master groups.

This subsection presents a comparative analysis of DFA α values derived from the right tibialis anterior, right lateral gastrocnemius, and right foot signals across the Control, Tai Chi, and Master groups. The box plots represent the distribution of the DFA α exponents, providing insights into neuromuscular control and gait variability associated with different training backgrounds. Figure 7a shows the DFA α values for the right tibialis anterior. The Control group exhibits a median α value of approximately 0.95, while the Tai Chi group’s median is similar at around 0.95 and the Master group shows a slightly higher median of approximately 1.00. The Master group also demonstrates reduced variability (interquartile range approximately 0.95–1.05) compared to the wider spread in the Control group (0.85–1.05), indicating more consistent neuromuscular activation patterns. A few minor outliers are present in all groups. Figure 7b presents the DFA α distribution for the right lateral gastrocnemius. Median α values are closely matched across groups at approximately 0.90–0.92. The spread remains relatively narrow for all groups (interquartile range roughly 0.85–1.00), suggesting that the calf muscle activation exhibits a similar long-range correlation structure regardless of training level. However, a small number of low α outliers are visible in the Control and Tai Chi groups. Figure 7c shows the DFA α values for the right foot based on FSR signals. The Control group displays a median α around 1.2, with the Tai Chi group also around 1.2 and the Master group slightly higher near 1.3. However, the variability in both Control and Tai Chi groups is much larger, with several extreme outliers reaching α values above 10 and below −5, indicating irregularities and inconsistencies in gait rhythm and foot–ground interaction. In comparison, the Master group shows a more compact spread (approximately 0.8–2.0), suggesting greater stride regularity and stability. Overall, these results indicate that the Master group tends to exhibit higher and more consistent DFA α values, particularly in the tibialis anterior muscle and foot–ground force signals, suggesting enhanced neuromuscular coordination and gait rhythmicity compared to the Control and Tai Chi groups.

### 4.6. ANOVA Comparison of DFA α Values Across Groups

**Figure 8:**
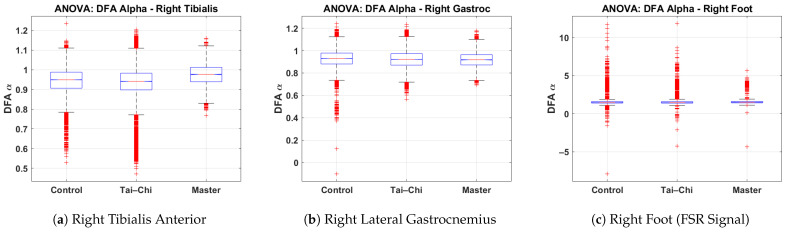
Group-wise comparison of DFA α values for the (**a**) right tibialis anterior, (**b**) right lateral gastrocnemius, and (**c**) right foot signals based on one-way ANOVA results across the Control, Tai Chi, and Master groups.

This subsection presents the results of a one-way ANOVA statistical analysis of the DFA α values derived from the right tibialis anterior, right lateral gastrocnemius, and right foot signals across the Control, Tai Chi, and Master groups. The box plots illustrate the distribution of DFA α values for each group, highlighting differences in neuromuscular control and gait regularity. Figure 8a presents the DFA α values for the right tibialis anterior muscle. The Control and Tai Chi groups show median α values around 0.95, while the Master group exhibits a slightly higher median of approximately 1.00. The spread is broader in the Control group (interquartile range roughly 0.85–1.05), whereas the Master group displays a tighter clustering, indicating more consistent neuromuscular activation. Several low outliers (below α≈0.7) are observed in the Control group. Figure 8b shows the DFA α distribution for the right lateral gastrocnemius. All three groups (Control, Tai Chi, and Master) demonstrate similar median values close to 0.90–0.92, with slightly narrower spreads compared to the tibialis anterior data. The Control group shows more low-value outliers (α below 0.6), which may reflect greater variability in calf muscle activation among untrained individuals. Figure 8c illustrates the DFA α values from the right foot FSR signal. The median values are around 1.2 for the Control and Tai Chi groups and slightly higher (near 1.3) for the Master group. However, a considerable number of extreme outliers (α>5 or α<−5) are present in all groups, particularly within the Control and Tai Chi groups. This wide variability suggests inconsistent gait force production and irregular foot–ground interactions in these groups, whereas the Master group tends to exhibit a narrower spread, indicating greater rhythmic stability. Overall, the ANOVA analysis supports the interpretation that advanced training such as that undertaken by the Master group is associated with more regular and stable gait and muscle activation patterns, as evidenced by higher and more consistent DFA α values. The one-way ANOVA yielded statistically significant group differences for the right tibialis anterior (*p* < 0.001) and right foot FSR (*p* < 0.001) and marginally significant differences for the right lateral gastrocnemius (*p* = 0.048). Post hoc multiple comparisons indicated that significant differences were primarily observed between the Master group and both the Control and Tai Chi groups, particularly for the tibialis anterior and FSR signals. These results confirm that higher levels of Tai Chi expertise are associated with more stable and consistent gait patterns, as reflected by elevated DFA α values.

To provide a group-level summary of the DFA α values obtained from all participants, Table 1 presents the mean and standard deviation of α for each signal and each group based on the full dataset under analysis.

## 5. Discussion

In the present study, we have investigated the application of cycle-wise detrended fluctuation analysis (DFA) to synchronized electromyography (EMG) and force-sensitive resistor (FSR) signals in order to characterise gait complexity, neuromuscular control, and stride-to-stride variability across individuals with differing levels of Tai Chi experience. Our analysis has focused on both muscle activation patterns and ground reaction force signals, allowing us to capture complementary aspects of the motor control system involved in human gait. The key finding of this study is that participants in the Master group (those engaged in long-term Tai Chi practice) exhibit significantly higher and more consistent DFA α values compared to the Control and intermediate Tai Chi practitioner groups. Higher DFA α exponents reflect increased long-range temporal correlations within the gait cycle, which are typically interpreted as signatures of more stable, regulated, and adaptable motor control systems. This is in agreement with previous research suggesting that complex motor training activities such as Tai Chi can enhance proprioceptive acuity, sensorimotor integration, and postural balance mechanisms. Among the recorded signals, the right tibialis anterior and right foot FSR signals demonstrated the most substantial group differences. The tibialis anterior plays a primary role in ankle dorsiflexion and foot clearance during gait, and its improved neuromuscular regulation in the Master group may contribute to smoother and more energy-efficient stride patterns. The FSR-derived foot pressure signals also demonstrated greater regularity in the Master group, suggesting a more rhythmic interaction between foot and ground, which further reflects stable gait patterns. Interestingly, the Tai Chi group, representing participants with moderate practice experience, showed DFA α values that generally fell in between those of the Control and Master groups, suggesting a possible progression in motor control adaptability with increasing Tai Chi experience. These findings may highlight the potential value of Tai Chi as a graded training intervention for improving dynamic balance and gait stability, and are particularly relevant to ageing populations and individuals at risk of falls. The use of cycle-wise DFA provides a unique nonlinear approach that extends beyond conventional linear variability metrics, allowing for more comprehensive evaluation of motor control complexity. By applying DFA to individual gait cycles, this method captures both intra-cycle dynamics and stride-to-stride fluctuations, which are particularly relevant when evaluating adaptive changes in neuromuscular control due to long-term motor practice. Furthermore, the combination of EMG and FSR modalities enabled simultaneous evaluation of central (neuromuscular) and peripheral (biomechanical) components of gait control, offering a holistic assessment framework. These results further support the potential of DFA-based analysis in movement science, rehabilitation monitoring, and sensor-based clinical gait assessment.

## 6. Conclusions

In conclusion, this study demonstrates the utility of detrended fluctuation analysis (DFA) as a robust nonlinear signal processing tool for assessing neuromuscular and gait stability adaptations in individuals with varying levels of Tai Chi expertise. The findings reveal that participants with advanced Tai Chi practice exhibit more regular and stable gait dynamics, as indicated by elevated and more consistent DFA α values derived from both EMG and FSR signals. Combined analysis of EMG and FSR data provided complementary information regarding both internal neuromuscular regulation and external gait force consistency, allowing for a comprehensive evaluation of gait complexity. The observed group-level differences suggest that long-term Tai Chi practice may contribute to improved sensorimotor integration, enhanced proprioceptive feedback, and more efficient control of lower limb musculature, ultimately promoting greater gait stability. These outcomes have potential implications for designing movement-based training programs targeting balance, stability, and fall prevention, particularly in ageing or clinical populations. The DFA-based framework introduced in this work may serve as a sensitive tool for monitoring intervention effects, detecting early neuromotor changes, and guiding rehabilitation strategies. Future research will focus on extending this analysis to larger participant cohorts, incorporating dual-task walking conditions, examining gait asymmetries, and exploring applications in real-world ambulatory monitoring using wearable sensor technologies.

## Figures and Tables

**Table 1:** Summary of DFA α values (mean ± SD) across participant groups.

Signal	Control	Tai Chi	Master
Right Tibialis Anterior	0.95±0.10	0.95±0.08	1.00±0.05
Right Lateral Gastrocnemius	0.90±0.07	0.92±0.06	0.93±0.05
Right Foot (FSR)	1.20±0.50	1.20±0.40	1.30±0.20

## Data Availability

The data used in this study are openly available as part of the Tai Chi Database (Version 1.0.2), hosted on PhysioNet: https://physionet.org/content/taichidb/1.0.2/. The dataset is distributed under the Open Data Commons Attribution License v1.0.

## References

[1] Scafetta N., Marchi D., West B.J. (2009). Understanding the complexity of human gait dynamics. Chaos Interdiscip. J. Nonlinear Sci..

[2] Verghese J., LeValley A., Hall C.B., Katz M.J., Ambrose A.F., Lipton R.B. (2006). Epidemiology of gait disorders in community-residing older adults. J. Am. Geriatr. Soc..

[3] Hausdorff J.M., Rios D.A., Edelberg H.K. (2001). Gait variability and fall risk in community-living older adults: A 1-year prospective study. Arch. Phys. Med. Rehabil..

[4] Figueiredo B., Frazão Á., Rouco A., Soares B., Albuquerque D., Pinho P. (2025). A Review: Radar Remote-Based Gait Identification Methods and Techniques. Remote Sens..

[5] Rana S.P., Dey M., Ghavami M., Dudley S. (2019). Non-contact human gait identification through IR-UWB edge-based monitoring sensor. IEEE Sens. J..

[6] Rana S.P., Dey M., Ghavami M., Dudley S. (2019). Signature inspired home environments monitoring system using IR-UWB technology. Sensors.

[7] Rana S.P., Dey M., Ghavami M., Dudley S. (2022). Markerless gait classification employing 3D IR-UWB physiological motion sensing. IEEE Sens. J..

[8] Rana S.P., Dey M., Ghavami M., Dudley S. (2021). 3-D gait abnormality detection employing contactless IR-UWB sensing phenomenon. IEEE Trans. Instrum. Meas..

[9] Rana S.P., Dey M., Ghavami M., Dudley S. ITERATOR: A 3D gait identification from IR-UWB technology. Proceedings of the 2019 41st Annual International Conference of the IEEE Engineering in Medicine and Biology Society (EMBC).

[10] Wayne P.M., Walsh J.N., Taylor-Piliae R.E., Wells R.E., Papp K.V., Donovan N.J., Yeh G.Y. (2014). Effect of Tai Chi on cognitive performance in older adults: Systematic review and meta-Analysis. J. Am. Geriatr. Soc..

[11] Li F., Harmer P., Fisher K.J., McAuley E., Chaumeton N., Eckstrom E., Wilson N.L. (2005). Tai Chi and fall reductions in older adults: A randomized controlled trial. J. Gerontol. Ser. A Biol. Sci. Med Sci..

[12] Su J.J., Lin R.S., Batalik L., Abu-Odah H., Pepera G., Xu Q., Yeung W.F. (2024). Effects of mind-body exercise on physical and psychosocial well-being of stroke patients: A systematic review and network meta-analysis. Geriatr. Nurs..

[13] Lage J.B., Nascentes G.A.N., Silva L.F., Borges M.C., Ferreira I.C.R., Lombardi L.A., Silva F.S., Espindula A.P. (2025). Electromyographic analysis of trunk and lower limb activation in children with cerebral palsy during gait and hippotherapy sessions. J. Bodyw. Mov. Ther..

[14] Bhongade A., Gupta R., Bhatia M., Prathosh A.P., Gandhi T.K. (2025). Classification of Gait Phases Using a Shank-Mounted Single IMU Sensor for Plane Level Walking. IEEE Sens. J..

[15] Peng C.K., Havlin S., Stanley H.E., Goldberger A.L. (1995). Quantification of scaling exponents and crossover phenomena in nonstationary heartbeat time series. Chaos Interdiscip. J. Nonlinear Sci..

[16] Goldberger A.L., Amaral L.A., Glass L., Hausdorff J.M., Ivanov P.C., Mark R.G., Mietus J.E., Moody G.B., Peng C.K., Stanley H.E. (2000). PhysioBank, PhysioToolkit, and PhysioNet: Components of a new research resource for complex physiologic signals. Circulation.

[17] Hausdorff J.M., Purdon P.L., Peng C.K., Ladin Z., Wei J.Y., Goldberger A.L. (1996). Fractal dynamics of human gait: Stability of long-range correlations in stride interval fluctuations. J. Appl. Physiol..

[18] Näf O.B., Bauer C.M., Zange C., Rast F.M. (2020). Validity and variability of center of pressure measures to quantify trunk control in stroke patients during quiet sitting and reaching tasks. Gait Posture.

[19] Wayne P.M., Kaptchuk T.J. (2008). Challenges inherent to t’ai chi research: Part I—t’ai chi as a complex multicomponent intervention. J. Altern. Complement. Med..

[20] Wu G. (2002). Evaluation of the effectiveness of Tai Chi for improving balance and preventing falls in the older population—A review. J. Am. Geriatr. Soc..

[21] Manor B., Costa M.D., Hu K., Newton E., Starobinets O., Kang H.G., Peng C., Novak V., Lipsitz L.A. (2010). Physiological complexity and system adaptability: Evidence from postural control dynamics of older adults. J. Appl. Physiol..

[22] Winter D.A. (1991). Biomechanics and Motor Control of Human Gait: Normal, Elderly and Pathological.

[23] Bamberg S.J.M., Benbasat A.Y., Scarborough D.M., Krebs D.E., Paradiso J.A. (2008). Gait analysis using a shoe-integrated wireless sensor system. IEEE Trans. Inf. Technol. Biomed..

[24] Mason R., Pearson L.T., Barry G., Young F., Lennon O., Godfrey A., Stuart S. (2023). Wearables for running gait analysis: A systematic review. Sports Med..

[25] Piryonesi S.M., Rostampour S., Piryonesi S.A. (2021). Predicting falls and injuries in people with multiple sclerosis using machine learning algorithms. Mult. Scler. Relat. Disord..

[26] Phinyomark A., Quaine F., Charbonnier S., Serviere C., Tarpin-Bernard F., Laurillau Y. (2013). EMG feature evaluation for improving myoelectric pattern recognition robustness. Expert Syst. Appl..

[27] Kirchner M., Schubert P., Liebherr M., Haas C.T. (2014). Detrended fluctuation analysis and adaptive fractal analysis of stride time data in Parkinson’s disease: Stitching together short gait trials. PLoS ONE.

[28] Kang H.G., Dingwell J.B. (2008). Effects of walking speed, strength and range of motion on gait stability in healthy older adults. J. Biomech..

[29] Lee Y., Kim G.B., Shin S. (2025). Association Between Lower Limb Strength Asymmetry and Gait Asymmetry: Implications for Gait Variability in Stroke Survivors. J. Clin. Med..

[30] Costa M., Goldberger A.L., Peng C.K. (2005). Multiscale entropy analysis of biological signals. Phys. Rev. E—Stat. Nonlinear Soft Matter Phys..

[31] Wayne P., Gow B., Hausdorff J., Peng C.K., Lipsitz L., Ahn A., Novak V., Manor B. (2021). Tai Chi, Physiological Complexity, and Healthy Aging-Gait. PhysioNet.

[32] Wayne P.M., Manor B., Novak V., Costa M.D., Hausdorff J.M., Goldberger A.L., Ahn A.C., Yeh G.Y., Peng C.K., Lough M. (2013). A systems biology approach to studying Tai Chi, physiological complexity and healthy aging: Design and rationale of a pragmatic randomized controlled trial. Contemp. Clin. Trials.

